# Supplementary material for: The structure of an archaeal oligosaccharyltransferase provides insight into the strict exclusion of proline from the N-glycosylation sequon
Source: Commun Biol. 2021 Aug 5;4:941. doi: 10.1038/s42003-021-02473-8 (PMC8342417; doi:10.1038/s42003-021-02473-8)
Supplement: Supplementary file 2 — Supplementary Information [file 42003_2021_2473_MOESM2_ESM.pdf]

Supplementary Information

The structure of an archaeal oligosaccharyltransferase provides insight into the strict exclusion of proline from the N-glycosylation sequon

Yuya Taguchi[1], Takahiro Yamasaki[1], Marie Ishikawa[1], Yuki Kawasaki[1], Ryuji Yukimura[1], Maki Mitani[1], Kunio Hirata[2], Daisuke Kohda[1]*

[1] Division of Structural Biology, Medical Institute of Bioregulation, Kyushu University, Fukuoka 812-8582, Japan

[2] RIKEN SPring-8 Center, 1-1-1 Kouto, Sayo, Hyogo 679-5148, Japan

* To whom corresponding should be addressed: E-mail: kohda@bioreg.kyushu-u.ac.jp.

Supplementary Table 1 Data Collection and Refinement Statistics

This is a revised version of the previously determined binary *Af*AglB-peptide complex (PDB: 5GMY). The position of the bound Mg^{2+} ion was corrected. The revised version of the coordinates has the same PDB entry name, 5GMY, using the entry versioning system. Values in parentheses are for the highest resolution shell.

	Binary complex of *Af*AglB and tethered peptide
**Data collection statistics**	
Beamline	SPring-8 BL44XU
Wavelength (Å)	0.9000
Oscillation range (°)	180
Space group	$P4_32_12$
Cell dimensions	$a = b = 121.5$ Å, $c = 181.3$ Å
Resolution range (Å)	49.90 – 3.50 (3.56 – 3.50)
Observed reflections	246049
Unique reflections	17649
Completeness (%)	99.8 (100.0)
$R_{merge}(I)$ [a]	0.106 (> 1)
$I / \sigma(I)$	44.7 (3.6)
**Refinement statistics**	
Resolution range (Å)	39.54 – 3.50 (3.63-3.50)
No. of reflections	17541(1669)
R_{work} [b]	0.213(0.356)
R_{free} [b]	0.283(0.441)
No. of non-hydrogen atoms	6887
Protein atoms	6886
Metal	1
rmsd [c] from ideal bond lengths (Å)	0.007
bond angles (°)	1.34
Ramachandran plot (%) [d]	
Favored region	83.9
Allowed region	13.3
Outlier region	2.8
Average B-factor (Å^2)	193.65
Protein atoms	193.66
Metal	135.75
No. of TLS groups[e]	2

[a] $R_{merge}(I) = (\Sigma_{hkl}\Sigma|I_i - \langle I \rangle|)/\Sigma_{hkl}\,\Sigma I_i$, where I_i is the intensity of the ith observation and $\langle I \rangle$ is the mean intensity. All sums over *hkl* extend only over unique reflections with more than one observation.

[b] $R_{work}/R_{free} = \sum_{hkl}|F_o - F_c|/\sum_{hkl}|F_o|$. R_{work} was calculated from the working set (94.9 % of the total reflections). R_{free} was calculated from the test set, using 5.1 % of the total reflections. The test set was not used in the refinement.

[c] rmsd, root mean square deviation.

[d] Calculated using the program *MOLPROBITY*.

[e] The TLS refinement was done with two TLS groups, consisting of the N-terminal TM region (residues 5-525 of chain A) and the C-terminal globular domain (residues 526-868 of chain A).

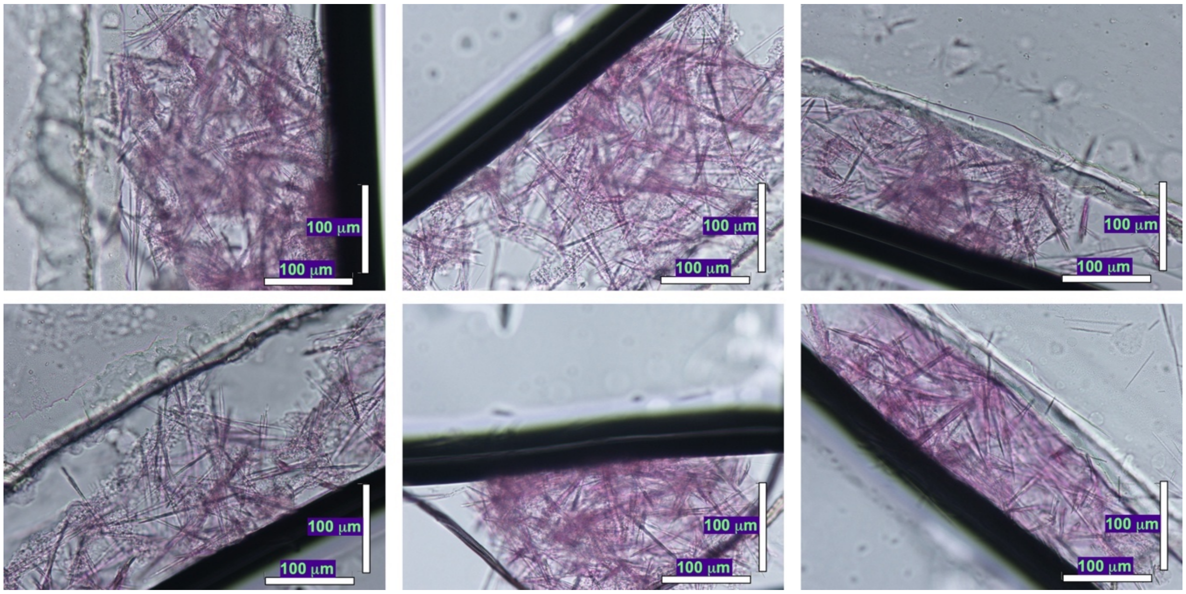

Supplementary Fig. 1 Microcrystals of the *Af*AglB protein obtained in a lipidic sponge mesophase. The crystals were easily identified by the magenta color derived from the TAMRA dye attached to the peptide.

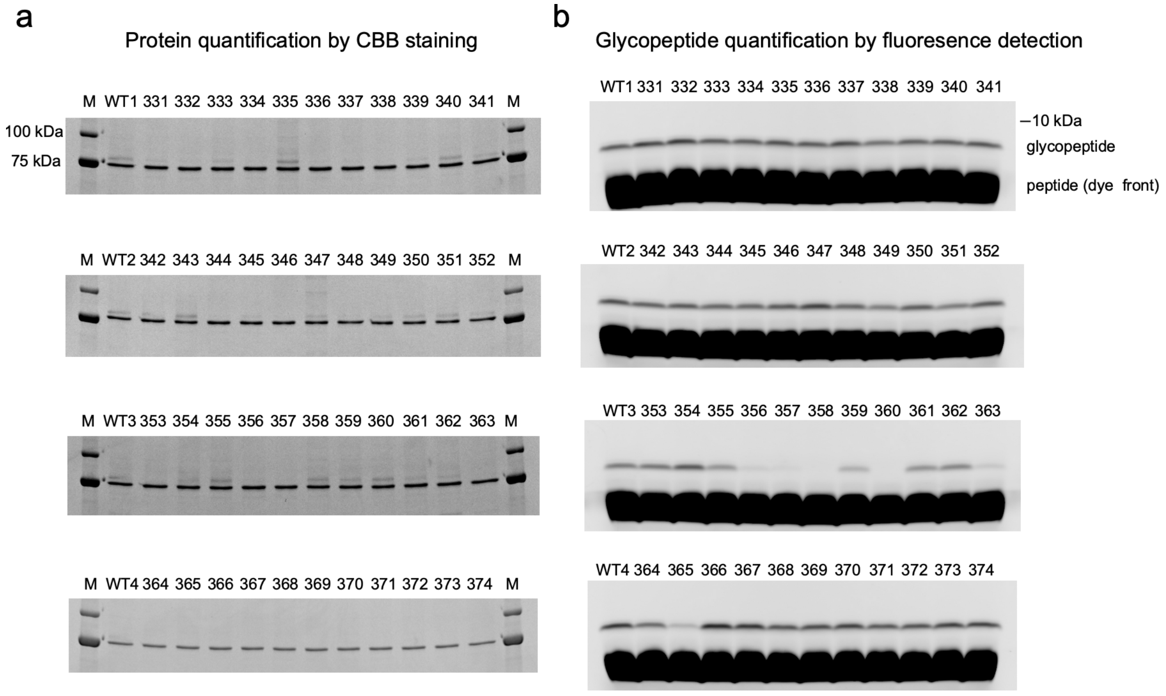

Supplementary Fig. 2 SDS-PAGE gel images in the alanine scanning mutagenesis of the EL5 loop. **a** Coomassie Brilliant Blue staining for protein quantification. **b** Fluorescence detection for glycopeptide quantification. In **a** and **b**, each representative PAGE image is shown from the respective triplicate experiments. The numbers indicate the residue positions of the amino acid mutations to Ala or Gly. The original data of Fig. 3. M, Molecular weight marker; WT, wild type. The migration positions of a 10-kDa marker protein and dye front are shown.

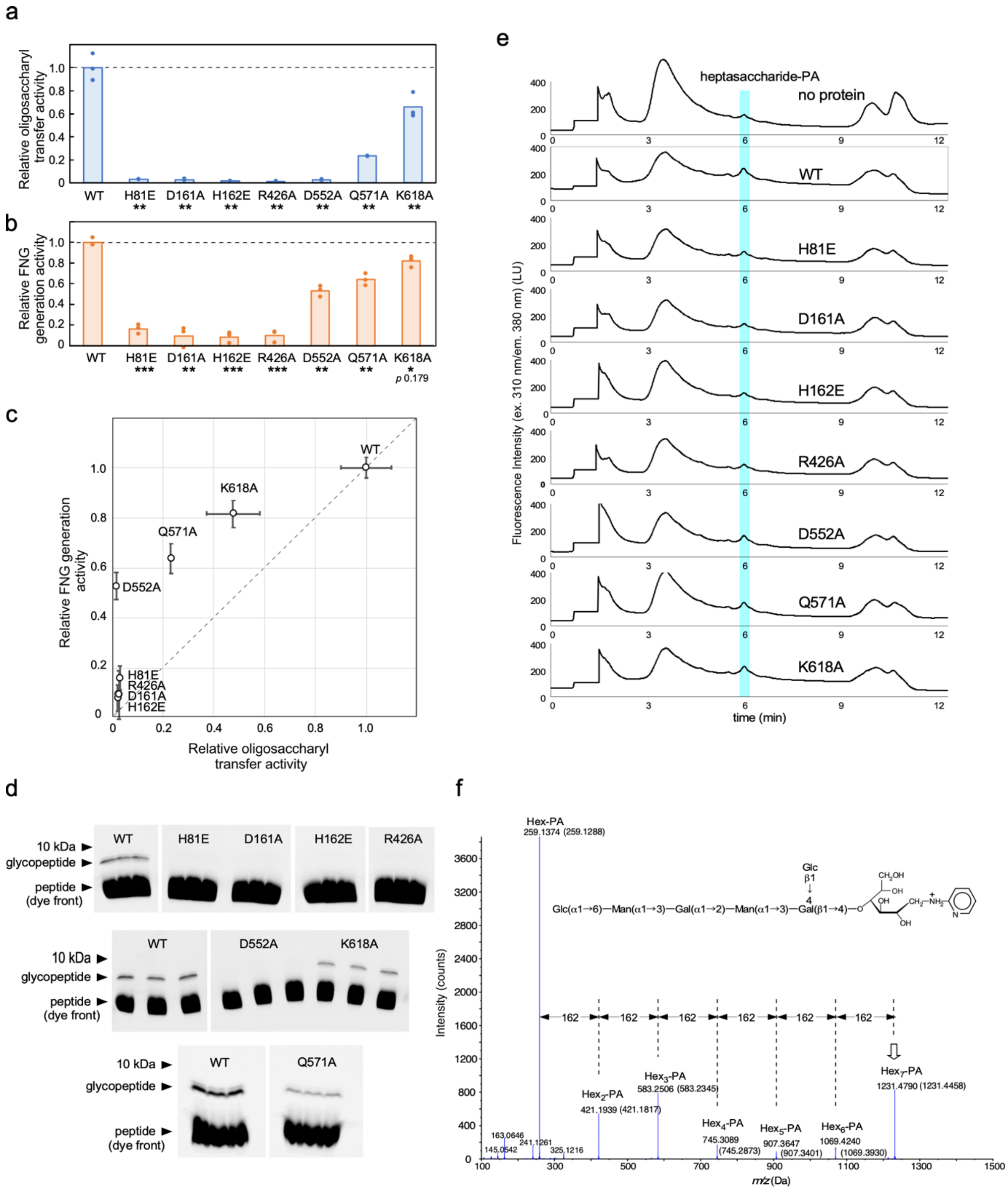

Supplementary Fig. 3 Correlation between the oligosaccharyl transfer and free N-glycan (FNG) generation activities of *Af*AglB mutants. a Oligosaccharyl transfer activity and **b** FNG generation activity of the *Af*AglB mutants. The bar heights indicate the mean values of triplicate measurements, and the error bars represent the standard deviation. Welch's two-sided t-test was applied between the

wild type and each mutant. The *p* values of 0.05 or less were considered statistically significant. $*p <$ 0.05, $** < 0.01$, $*** < 0.001$. **c** Correlation plot between the oligosaccharyl transfer and FNG generation activities. Data from **a** and **b**. **d** Fluorescence detection for the glycopeptide quantification. The original data of **a**. Three independent SDS-PAGE analyses were run for three groups: (H81E, D161A, H162E, R426A), (D552A, K618A), and (Q571A). Triplicate measurements were done for each mutant. In each run, the band intensity of WT was used to normalize the amount of glycopeptide production. The migration positions of a 10-kDa marker protein and dye front are shown. **e**, UPLC chromatograms for FNG quantification. The original data of **b**. Pyridylaminated (PA) FNGs were eluted from the normal-phase UPLC column around 6.0 min. The broad peaks were the mixture of the hepta- and octasaccharide-PAs, with one optional sulfate group on the reducing-end monosaccharide residue. The analysis of the reaction mixture without the *Af*AglB protein revealed the non-enzymatic generation of FNG (*top* chromatogram). The amounts of the enzymatically generated FNG were calculated by subtraction of the peak area of the non-enzymatically generated FNG. **f** ESI-MS/MS spectrum of the FNG-PA, illustrated by an example of the pyridylaminated hexasaccharide (Hex_7-PA). The UPLC system was directly connected to a mass spectrometer QSTAR Elite (ABSciex) operated in the positive ion mode. The precursor ion is marked by the vertical arrow. The *inset* shows the chemical structure of the Hex_7-PA. The expected *m/z* values were observed within 0.03 of the theoretical mass values (in parentheses).

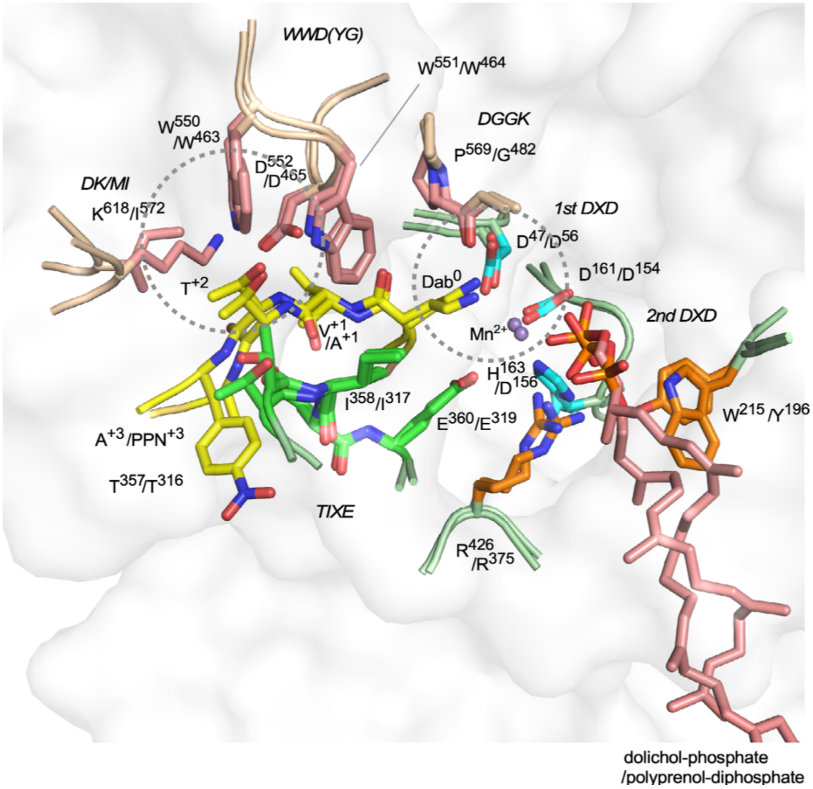

Supplementary Fig. 4 Superimposition of the ternary *Af*AglB and *Cl*PglB (PDB: 6GXC) complex structures. Amino acid residues related to the sequon recognition, and the catalytic function are shown and labeled. The two structures were superimposed by the PyMOL align command, using all heavy atoms belonging to the 3-residue sequon and 4-residue TI*X*E motif, the conserved three acidic residues (D^{47}/D^{56}, D^{161}/D^{154}, and H^{163}/D^{156}) in the two D*X*D motifs, the manganese ion, and the phosphate group directly linked to the oligosaccharide. The rms distance is 0.56 Å for 43 pairs of atoms. The /-delimited residue names represent the amino acid residue of *Af*AglB (first) and that of *Cl*PglB (second). The sequon peptides containing the Dab residues are shown as yellow sticks, and the dolichol-phosphate molecule derived from the natural *Af*LLO, and the polyprenol-diphosphate part of the *Cl*LLO analog are shown as salmon sticks. The GlcNAc residue of *Cl*LLO is not displayed for clarity. The manganese ions are shown as purple spheres. The previously identified interactions between the *Af*AglB/*Cl*PglB proteins and the side-chain groups of Asn^{0}/Dab^{0} and Thr^{+2}/Thr^{+2} are shown enclosed in dotted circles. Note that the resolution of the coordinates of PDB: 6GXC is 3.4 Å, but the positional accuracy of atoms is expected to be as high as that of *Af*AglB (2.7 Å) because the structure of the 6GXC was solved by the molecular replacement method, using the PDB entry 5OGL (2.7 Å) as the template.

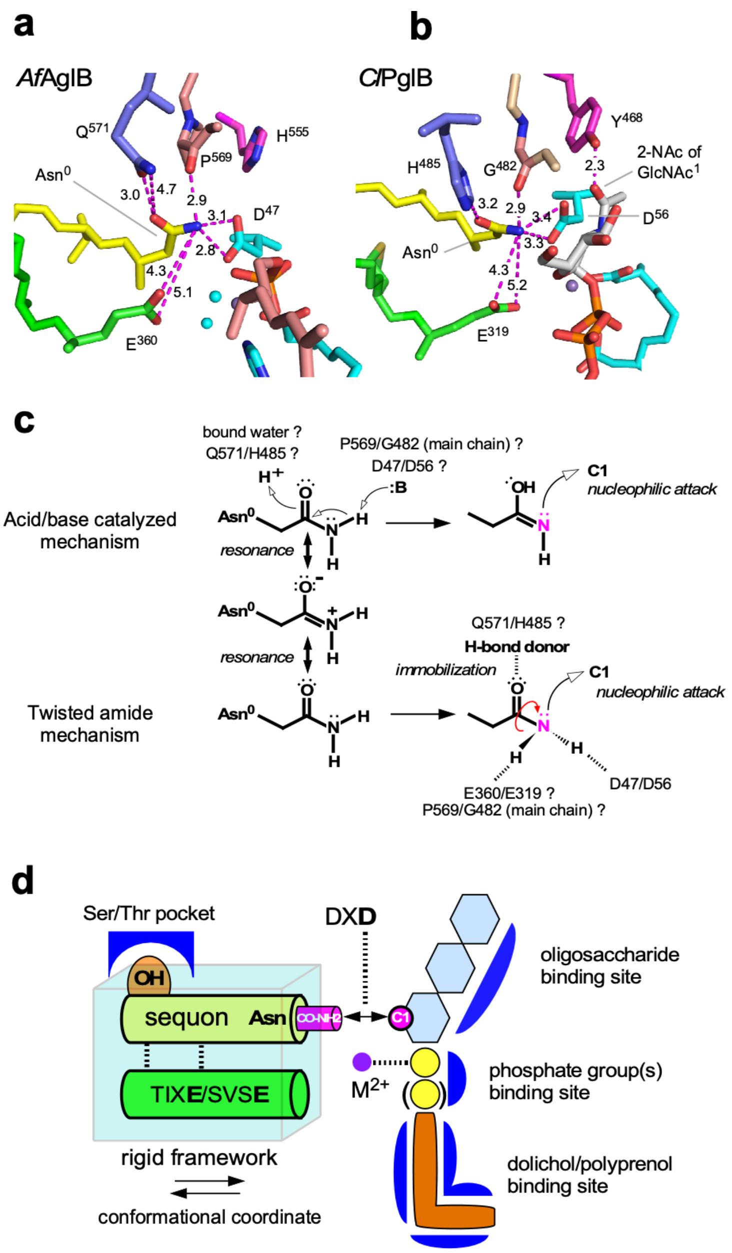

Supplementary Fig. 5 Hypothetical mechanisms for the catalytic activation of the amide nitrogen. Amino acid residues around the side-chain carboxamide group of the acceptor Asn in **a** *Af*AglB, and **b** *Cl*PglB (PDB: 6GXC). In **a** and **b**, the side-chain amino group of the Dab residue was displaced with the carboxamide group to indicate the position of the carbonyl group. The numbers

are the interatomic distances in Å units. **c** Two previously proposed activation mechanisms. The amide nitrogen of a carboxamide group is poorly reactive due to electron delocalization from the amine to the carbonyl group, indicated by resonance. The amide nitrogen could be activated by an acid/base catalysis; *i.e.*, protonation of the carbonyl oxygen and deprotonation of the amide nitrogen, or amide twisting; *i.e.,* rotation around the N–C bond by the formation of two hydrogen bonds with the conserved acidic residues. **d** Supportive mechanism that compensates for the poor nucleophilicity of the amide nitrogen. The rigid sequon-TI*X*E/SVSE structure restricts the motion of the side chain of the acceptor Asn in only one direction, for the effective conversion of energy from the conformational to chemical coordinates and forces the nitrogen atom to move closer to the C1 carbon of the LLO within a reactive distance in the transition state.

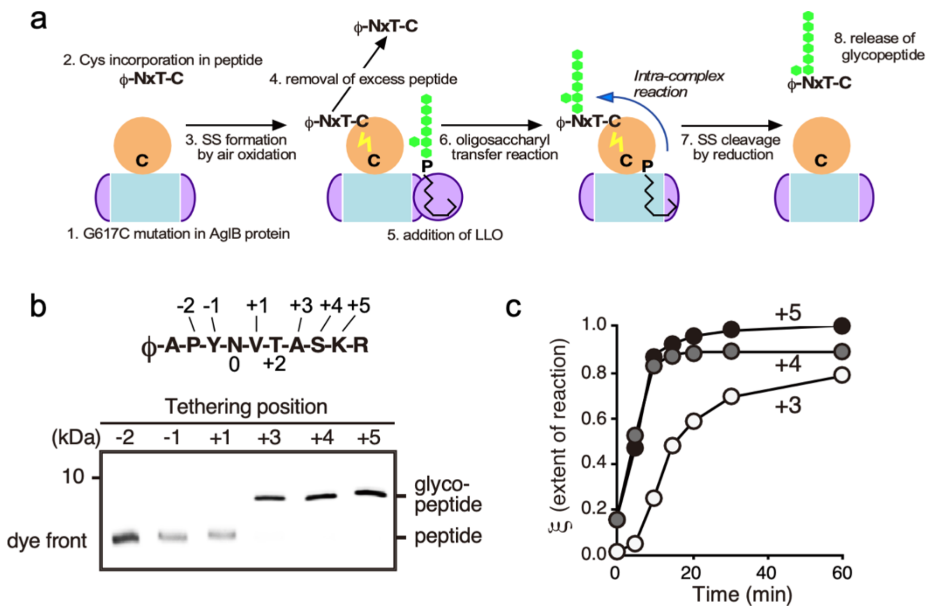

Supplementary Fig. 6 Preparation of a catalytically competent cross-linked *Af*AglB complex. a Outline of the preparation procedure. The *Af*AglB protein is a single-subunit membrane polypeptide containing 13 transmembrane helices in the N-terminal region (cyan rectangle) embedded in the DDM detergent micelles (purple semi ovals). A single cysteine is introduced at position 617 in the C-terminal globular domain (orange circle) (step 1). The purified G617C mutant is mixed with an acceptor peptide containing the N-glycosylation sequon (NxT), a Cys residue at various positions for disulfide tethering, and a fluorescent TAMRA dye (ϕ) attached to the N-terminus for detection (step 2). An intermolecular disulfide bond is formed by air oxidation (step 3), and then unreacted peptides are removed by membrane filtration or dialysis (step 4). At this stage, it is ready to use for crystallization. The addition of the donor substrate, *A. fulgidus* LLO solubilized in DDM (step 5), initiates the oligosaccharyl transfer reaction to generate the glycopeptide on the *Af*AglB protein (step 6). The addition of a reducing reagent, DTT, cleaves the disulfide bond (step 7), to release the glycopeptide product (step 8). **b** The reaction mixture containing the crosslinked *Af*AglB─peptide complex and *Af*LLO was incubated at 65°C. After an incubation at room temperature in the presence of DTT, the released peptide and glycopeptide were separated on SDS─PAGE gels and quantified by in-gel fluorescence imaging. The migration positions of a 10-kDa marker protein and dye front are shown. **c** Variable efficiencies of the intra-molecular oligosaccharyl transfer reactions with different tethering positions. The ξ value was calculated according to the equation ξ = (“fluorescence intensity of glycopeptide”)/(“fluorescence intensity of glycopeptide” + “fluorescence intensity of unreacted peptide”).

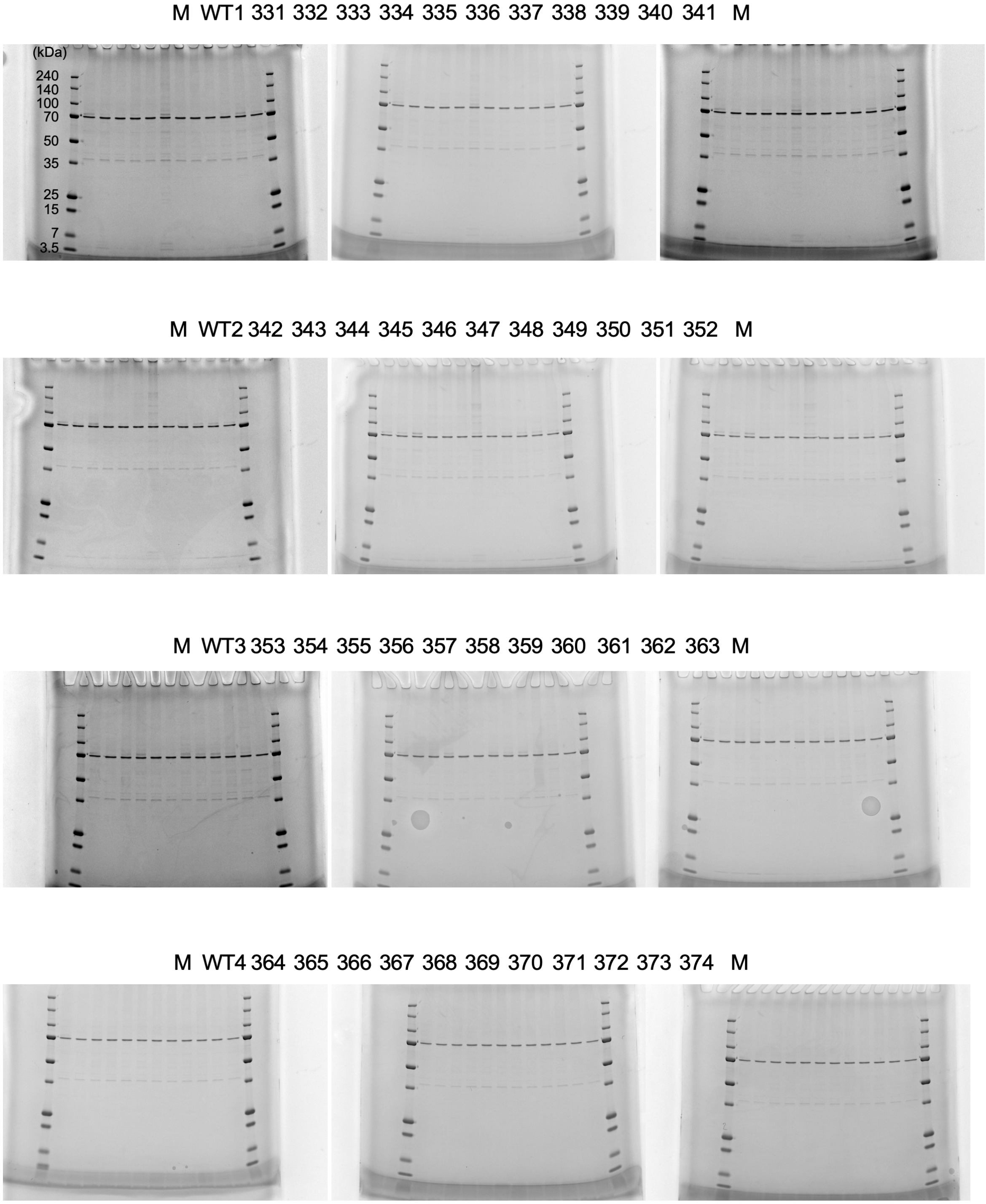

Supplementary Fig. 7 Unprocessed CBB stained gel images for protein quantification. The original data of Supplementary Fig. 2a.

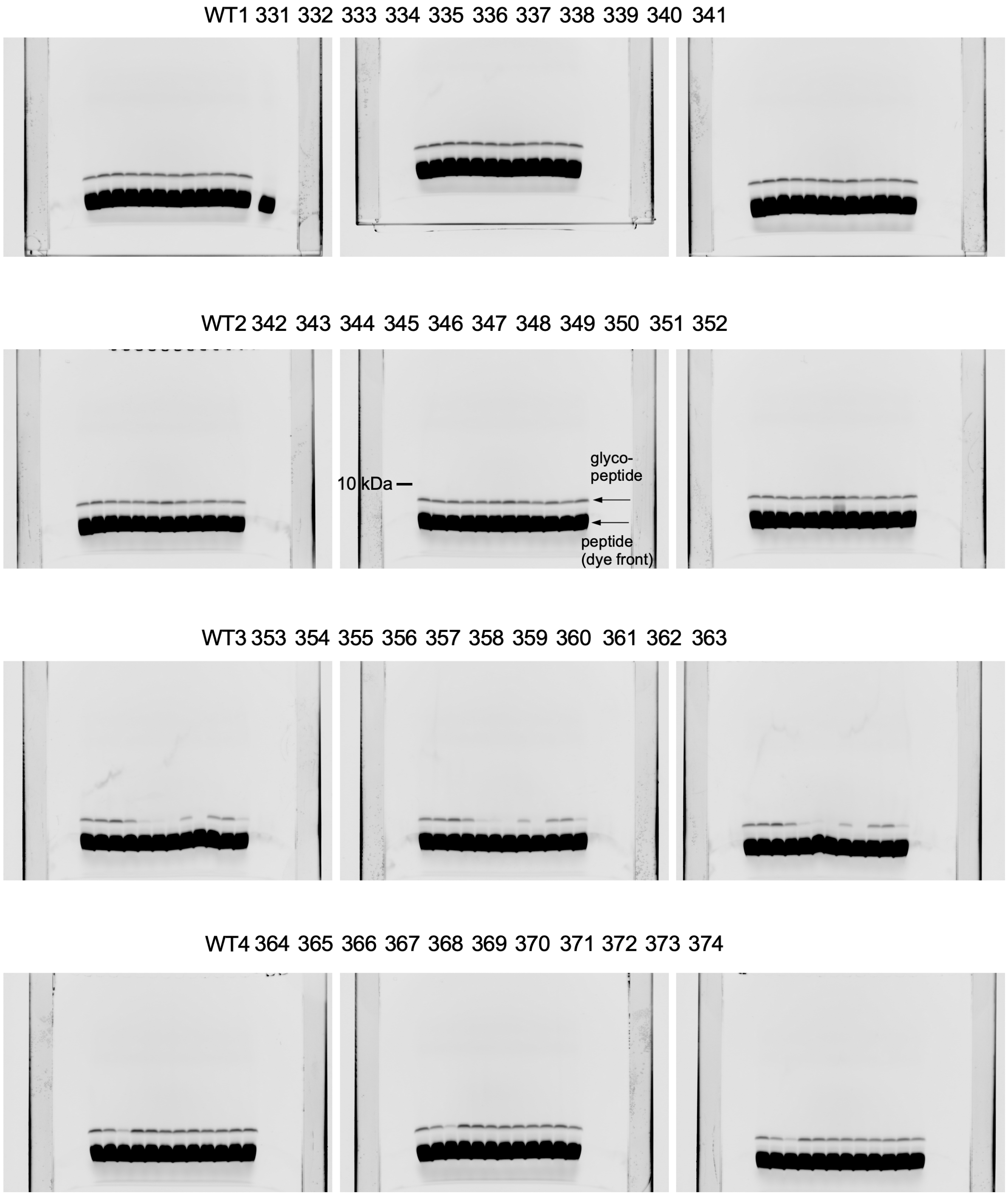

Supplementary Fig. 8 Unprocessed gel images of fluorescent detection for glycopeptide quantification. The original data of Supplementary Fig. 2b. The migration positions of a 10-kDa marker protein and dye front are shown.

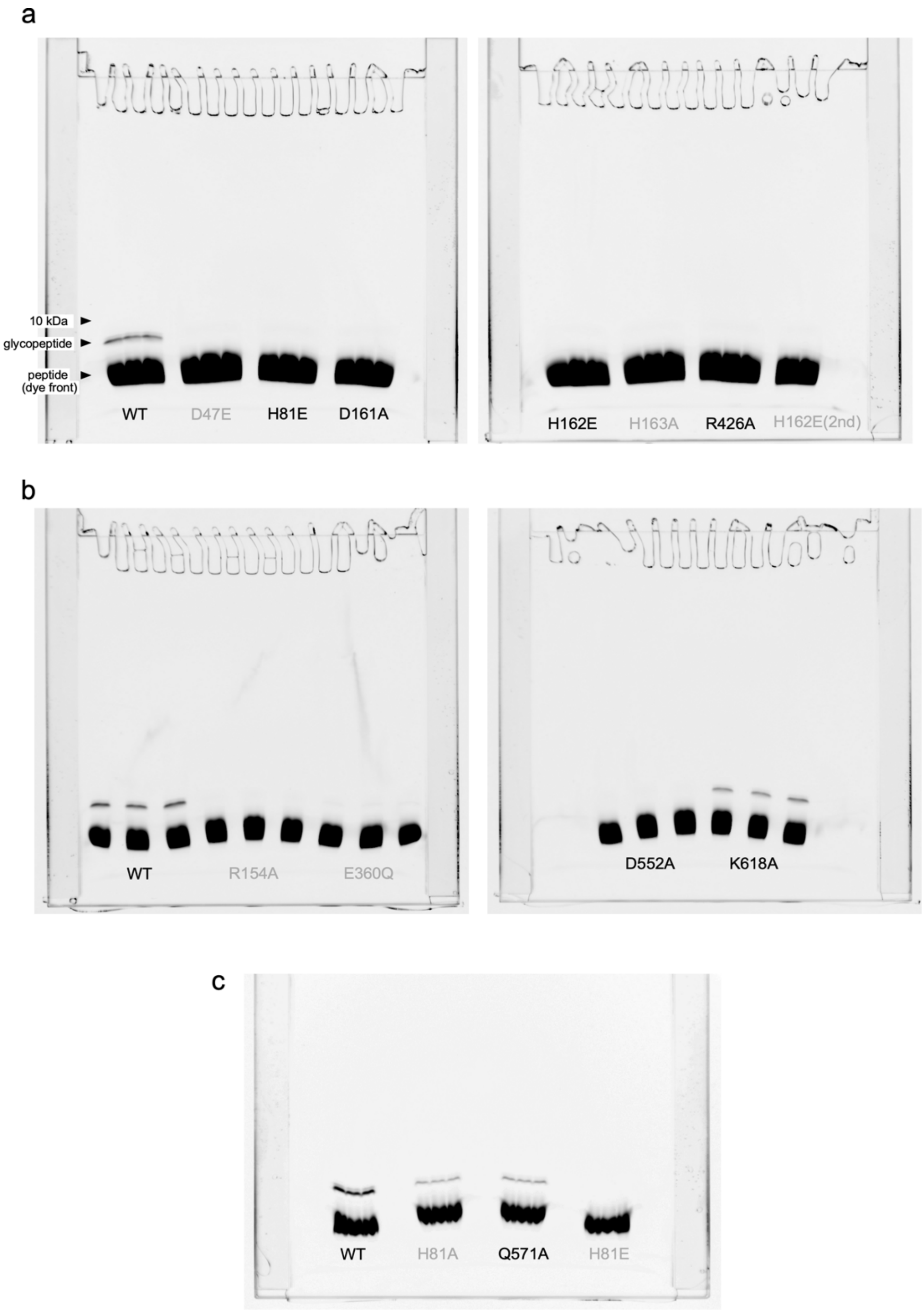

Supplementary Fig. 9 Unprocessed gel images of fluorescent detection for glycopeptide quantification. The original data of Supplementary Fig. 3d. The migration positions of a 10-kDa marker protein and dye front are shown. Gel electrophoresis labeled a, b, and c were performed on different days.

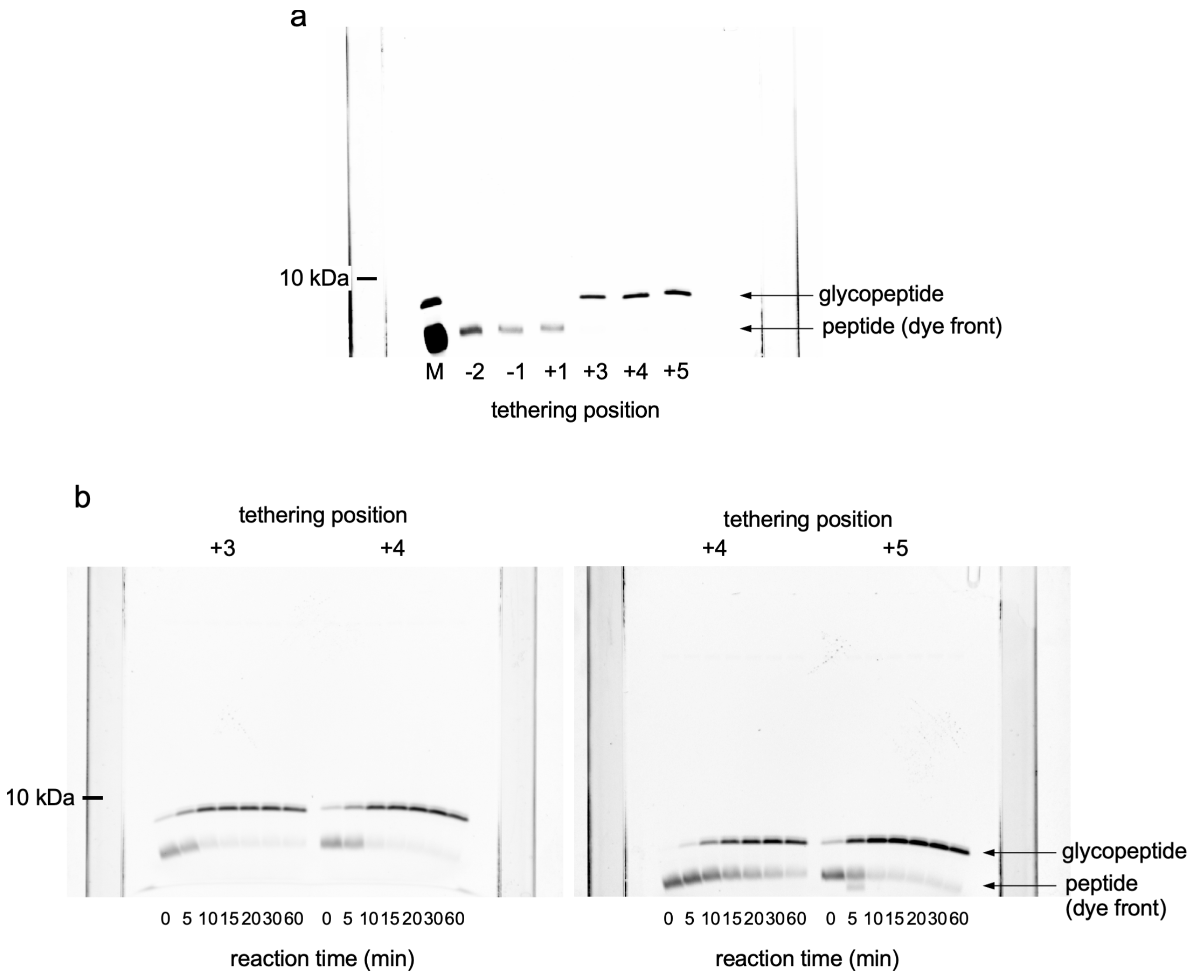

Supplementary Fig. 10 Unprocessed gel images of fluorescent detection for glycopeptide quantification. a The original data of Supplementary Fig. 6b. **b** The original data of Supplementary Fig. 6c. The migration positions of a 10-kDa marker protein and dye front are shown.
