# Supplementary material for: The structure of an archaeal oligosaccharyltransferase provides insight into the strict exclusion of proline from the N-glycosylation sequon
Source: Commun Biol. 2021 Aug 5;4:941. doi: 10.1038/s42003-021-02473-8 (PMC8342417; doi:10.1038/s42003-021-02473-8)
Supplement: Supplementary file 3 — Description of Additional Supplementary Files [file 42003_2021_2473_MOESM3_ESM.pdf]

**Description of Additional Supplementary Files**

File Name: Supplementary Data 1
Description: Source data of Fig 3 (Excel) Oligosaccharyl transfer assay data of alaninescanning mutagenesis

File Name: Supplementary Data 2
Description: Source data of Fig 4 (Excel) Ramachandran plots

File Name: Supplementary Data 3
Description: Source data of Supplementary Fig 3a and 3b. (Excel) Oligosaccharyl transfer assay and FNG generation assay data of AfAglB mutants

File Name: Supplementary Data 4
Description: Source data of Supplementary Fig 6c (Excel) Time course data of the oligosaccharyl transfer assays
