# Supplementary material for: The structure of an archaeal oligosaccharyltransferase provides insight into the strict exclusion of proline from the N-glycosylation sequon
Source: Commun Biol. 2021 Aug 5;4:941. doi: 10.1038/s42003-021-02473-8 (PMC8342417; doi:10.1038/s42003-021-02473-8)
Supplement: Supplementary file 8 — reporting summary [file 42003_2021_2473_MOESM8_ESM.pdf]

nature research

Corresponding author(s): Daisuke Kohda

Last updated by author(s): Jul 14, 2021

Reporting Summary

Nature Research wishes to improve the reproducibility of the work that we publish. This form provides structure for consistency and transparency in reporting. For further information on Nature Research policies, see our Editorial Policies and the Editorial Policy Checklist.

Statistics

For all statistical analyses, confirm that the following items are present in the figure legend, table legend, main text, or Methods section.

n/a	Confirmed	
☐	☒	The exact sample size (n) for each experimental group/condition, given as a discrete number and unit of measurement
☐	☒	A statement on whether measurements were taken from distinct samples or whether the same sample was measured repeatedly
☐	☒	The statistical test(s) used AND whether they are one- or two-sided<br>*Only common tests should be described solely by name; describe more complex techniques in the Methods section.*
☐	☒	A description of all covariates tested
☐	☒	A description of any assumptions or corrections, such as tests of normality and adjustment for multiple comparisons
☐	☒	A full description of the statistical parameters including central tendency (e.g. means) or other basic estimates (e.g. regression coefficient) AND variation (e.g. standard deviation) or associated estimates of uncertainty (e.g. confidence intervals)
☐	☒	For null hypothesis testing, the test statistic (e.g. F, t, r) with confidence intervals, effect sizes, degrees of freedom and P value noted<br>*Give P values as exact values whenever suitable.*
☒	☐	For Bayesian analysis, information on the choice of priors and Markov chain Monte Carlo settings
☒	☐	For hierarchical and complex designs, identification of the appropriate level for tests and full reporting of outcomes
☒	☐	Estimates of effect sizes (e.g. Cohen's d, Pearson's r), indicating how they were calculated

Our web collection on statistics for biologists contains articles on many of the points above.

Software and code

Policy information about availability of computer code

Data collection	ZOO(beamline control software at SPring-8 BL32XU), BSS(SPring-8 standard beamline control software at SPring-8)
Data analysis	KAMO(SPring-8 automatic data-processing software), XDS(X-ray data indexing and integration), XSCALE(X-ray data scaling and merging), POINTLESS(X-ray data processing, symmetry deduction), CTRUNCATE(X-ray data processing, intensity to amplitude conversion and data statistics), BLEND(X-ray data processing, hierarchical clustring), phenix.phaser(molecular replacement), PHENIX(structure refinement), REFMAC5(structure refinement), Coot(atomic modeling), eLBOW(generation of the CIF restraints of ligands), CHARM-GUI(generation of the coordinates of lipid bilayer) , RAMPAGE(generation of Ramachandran plots), PyMOL(molecular graphics and figure generation)

For manuscripts utilizing custom algorithms or software that are central to the research but not yet described in published literature, software must be made available to editors and reviewers. We strongly encourage code deposition in a community repository (e.g. GitHub). See the Nature Research guidelines for submitting code & software for further information.

Data

Policy information about availability of data

All manuscripts must include a data availability statement. This statement should provide the following information, where applicable:

- Accession codes, unique identifiers, or web links for publicly available datasets
- A list of figures that have associated raw data
- A description of any restrictions on data availability

Atomic structure and its structure factors have been deposited in the Protein Data Bank (PDB) with an accession code, 7E9S. The source data underlying Fig. 3, Fig. 4, Supplementary Fig. 3a and b, and Supplementary Fig. 6c are provided as Supplementary Data online.The uncropped gel images related to Fig. 2a, Fig. 2b, Supplementary Fig. 3d, and Supplementary Fig. 6d are provided as Supplementary Figures.

Field-specific reporting

Please select the one below that is the best fit for your research. If you are not sure, read the appropriate sections before making your selection.

[x] Life sciences [ ] Behavioural & social sciences [ ] Ecological, evolutionary & environmental sciences

For a reference copy of the document with all sections, see nature.com/documents/nr-reporting-summary-flat.pdf

Life sciences study design

All studies must disclose on these points even when the disclosure is negative.

Sample size	Small-wedge X-ray diffraction data were collected from 2,500 microcrystals.
Data exclusions	Some of the small-wedge X-ray diffraction data were excluded based on the clustering by unit-cell parameters in the program KAMO. Structure factors were truncated based on I/sigma > 1 and CC1/2 > 50 % generated by the program XSCALE.
Replication	Oligosacchary transfer assay (except for the assay in Supplementary Fig. S6b) and FNG generation assay were replicated in three independent experiments. Representative gel images are presented in the main text and Supplementary information. Time course of the generation of the glycopeptide product was followed instead of replication in Supplementary Fig. S6b.
Randomization	5% of the structure factors was assigned to the Rfree set randomly by the program FREEFLAG(CCP4).
Blinding	Blinding was not used in this study.

Reporting for specific materials, systems and methods

We require information from authors about some types of materials, experimental systems and methods used in many studies. Here, indicate whether each material, system or method listed is relevant to your study. If you are not sure if a list item applies to your research, read the appropriate section before selecting a response.

Materials & experimental systems

n/a	Involved in the study
[x]	[ ] Antibodies
[x]	[ ] Eukaryotic cell lines
[x]	[ ] Palaeontology and archaeology
[x]	[ ] Animals and other organisms
[x]	[ ] Human research participants
[x]	[ ] Clinical data
[x]	[ ] Dual use research of concern

Methods

n/a	Involved in the study
[x]	[ ] ChIP-seq
[x]	[ ] Flow cytometry
[x]	[ ] MRI-based neuroimaging
